# The role of innovation and entrepreneurship employee training programs in enhancing organizational commitment from the perspective of industry–education integration

**DOI:** 10.3389/fpsyg.2025.1527741

**Published:** 2025-02-21

**Authors:** Zijing Wu, Qiang Li, Biao Zhang

**Affiliations:** Guangzhou College of Technology and Business, Guangzhou, China

**Keywords:** employee training, organizational commitment, job satisfaction, career growth, thematic analysis, employee engagement, qualitative research, knowledge sharing

## Abstract

This study aims to explore the role of employee training programs in enhancing organizational commitment. In the context where organizational commitment is crucial for enterprises’ stable development and employee training programs are an important part of human resources development, understanding their relationship is of great significance. Qualitative research methods were employed. Specifically, semi - structured interviews were carried out with employees from diverse industries. Then, thematic analysis was used to analyze the interview data. Through the thematic analysis, several key themes emerged. The relevance of training content to employees’ job roles and career aspirations has a significant impact on training effectiveness. Employees highly value practical and work - applicable training, which is closely linked to job satisfaction. The quality of trainers is also crucial, as competent and engaging trainers can enhance the perceived value of training. Moreover, knowledge sharing promoted by training programs benefits employees’ personal and professional development and strengthens the organization’s collaborative culture. The results highlight that these findings have practical implications for managers. They should design training programs that match employees’ needs and career goals, invest in improving trainers’ capabilities, and create a knowledge - sharing - friendly environment. By taking these actions, managers can enhance employees’ job satisfaction, skills, and career growth, thereby strengthening organizational commitment. Looking ahead, future research could conduct longitudinal studies to track the long - term effects of training on organizational commitment. Also, exploring the influence of organizational culture and individual differences on training outcomes can offer more targeted training strategies for different organizations and employees.

## 1 Introduction

In addition to other components of organizational strategy, employee training programs are central to employee development, which will help improve employees’ performance and increase long-term commitment to the organization (Al-Aali, 2021; Hasan and Chowdhury, 2023) however, in the fast-changing business world, where technologies are developing at a fast pace and markets are also becoming global, organizations are becoming increasingly dependent on training programs to achieve a competitive edge and keep top talent. In addition to providing technical training, training in such initiatives involves not only the technical skills of the employees being worked upon but also helping employees with soft skills, such as leadership, communication, and problem-solving skills (Shahriari et al., 2023). With a growing emphasis on the human capital that constitutes firms, training programs, more generally, and the inputs and outputs within them, have begun to face more intensive scrutiny about the link between organizational outcomes (e.g., productivity, employee satisfaction, commitment) and the presence or success of training.

In this study, organizational commitment is defined as the psychological state of an employee, referring to an employee’s relationship with his or her organization, encompassing emotional attachment, perceived costs of leaving, and a sense of obligation to stay. This concept has been widely studied in the management and organizational behavior literature (Giao et al., 2020) proposed affective, continuance, and normative commitment. Affective commitment is being emotionally attached to the organization, continuance commitment is the perceived cost of leaving the organization, and normative commitment is a feeling of obligation to stay. Each dimension is important in detailing the employee’s commitment to the organization. Many factors influence organizational commitment, such as compensation, work–life balance, and organizational culture (Cherif, 2020). Among them, employee training programs have been at the forefront in driving commitment. When organizations offer chances or potential for their employees to grow and develop skills, they become more patient and loyal to the organization because of their sense of value and belonging (Ridwan et al., 2020). Second, training programs signal to employees that the organization is interested in their future and will help them develop skills to flourish in that position and for a long future with the organization. This intersection between training programs and organizational commitment has been studied in this research, with this study investigating how employees perceive the effects of training on employees’ organizational commitment.

However, even though training is recognized as important, many organizations have trouble achieving the fuller potential of their training initiatives. One key problem is that training programs might be well designed to develop employment skills but not linked to the greater goal of increasing organizational commitment. In some cases, training programs can be triggered by employees taking part in training (Akla and Indradewa, 2022). They may not see the actual benefit of the training programs for career progression or development of the employee, leading to the training programs being ineffective in perpetuating deeper attachment with the organization. Consequently, a significant gap still exists in the understanding of how these training programs are perceived and experienced by employees themselves instead of quantitatively (loyalty, emotional attachment, etc.) Over the past few years, there has been increasing awareness of the need to move beyond the quantitative aspects of training programs and their impact on employee behavior. Although a variety of quantitative studies have been conducted to examine the connection between training and commitment, only a few studies have used a qualitative approach to address the extended meaning of commitment: to discover the lived experiences of employees and the different factors involved in their commitment. Not capturing all employees’ views on how training impacts their commitment overlooks information that the organization can use for more effective training program design and delivery (Mahmood Aziz et al., 2021). This research endeavors to fill these gaps by exploring how employees perceive the role of training programs in relation to organizational commitment. Specifically, it examines how employees subjectively experience the training they receive at work and how they view these experiences in connection with their emotional and behavioral commitment to the organization. Using a qualitative approach, this study offers a richer, deeper understanding of how training programs indirectly contribute to organizational commitment, a point of vital interest both from the academic and practitioner’s perspective.

This research aims at the overall level to examine the impact of employee training programs on employees’ organizational commitment. This study attempts to discover how training programs affect employees’ subjective and perceptual commitment to the organization by focusing on employees’ experiences. For several reasons, this study is significant. It first fills a critical gap in the literature by examining the qualitative aspects of employee training programs and their effects on organizational commitment. Furthermore, although there is a large body of research regarding the relationship between training and commitment, much of it is grounded in quantitative work, which does not explain the complexity of employees’ experiences and perceptions of training. Through a qualitative approach, this study offers in-depth insight into how training programs shape employees’ commitment to their organizations to improve their training strategies. Second, this research has practical implications for organizations. In an increasingly cutthroat work environment, retaining employees is an issue of great concern for many organizations, making it crucial to understand the factors contributing to organizational commitment to develop effective human resource strategies. Company training programs are perceived as powerful tools for improving employee satisfaction and engagement; however, they do not have a clear positive effect on commitment. This study explores how training programs influence employees’ commitment to the organization. It will help organizations with actionable news on what should and should not be included in training programs to help organizations better attain their commitment-building objectives.

This study is also relevant for policymakers and HR professionals who implement and design employee training programs. This research will generate insights to help inform policies to retain and develop employees. Specifically, by concentrating on qualitative training programs, the study will offer a wider appreciation of how such programs may be designed to engender skill acquisition and deeper emotional and behavioral commitment to the firm. This study has the potential to contribute to the field of organizational behavior more generally by advancing our understanding of the relationship between employee development and organizational commitment. Most of the literature addresses individual-level training outcomes, such as job performance and satisfaction, and this study is intended to identify organizational-level training outcomes specifically in terms of employee commitment. This will provide a much larger overview of the importance of training programs for organizational success and sustainability.

## 2 Literature review

### 2.1 Theories of organizational commitment

#### 2.1.1 Affective committee

Organizational commitment is a concept that frequently emerges in the literature on management and organizational behavior (Guzeller and Celiker, 2020). Sumarsi and Rizal (2021) noted that organizational commitment consists of three distinct components: continuance commitment, normative commitment, and affective commitment. Affective commitment refers to the emotional attachment of employees to the organization. Demır (2020) argued that employees with a strong and effective commitment to the organization identify with the organization’s goals and values, have a sense of belonging, and are more likely to exert efforts in favor of the organization. This type of commitment is formed when employees perceive that there are congruences between themselves and the organization’s objectives and receive feedback in the form of being valued. Numerous studies have demonstrated a strong positive correlation between affective commitment and various favorable organizational outcomes, including higher job satisfaction, reduced turnover rates, and improved job performance (Abdirahman et al., 2020).

#### 2.1.2 Continance commitment

On the other hand, continuance commitment is driven by employees’ awareness of the costs associated with leaving the organization (Astuty and Udin, 2020). Employees with a strong continuance commitment stay in the organization because they believe that leaving leads to significant personal or financial losses, such as the loss of retirement benefits, long-term job security, or career advancement opportunities (Cherif, 2020). Although this type of commitment is often associated with a lower level of employee satisfaction than affective commitment is, it still plays a crucial role in reducing turnover. However, some researchers believe that employees who stay due to obligation or fear of loss may not be as engaged or productive as those who stay due to emotional attachment (Purwanto, 2020).

#### 2.1.3 Normative commitment

Normative commitment refers to the sense of obligation of employees to stay in the organization. This sense of obligation usually comes from the socialization process. For example, family members, teachers, or mentors teach them to attach importance to the sense of responsibility toward employers. Employees with such a normative commitment believe that they must stay in the organization for moral or ethical reasons, such as having received training, help, or development in the organization (Chahar et al., 2021). Researchers have noted that organizations can build normative commitment by providing employees with development opportunities, and in return, employees generate a sense of loyalty and responsibility (Jamiiaturochmah et al., 2021).

#### 2.1.4 The role and limitations of the three-components model of organizational commitment

The three-component model of organizational commitment provides a complete framework for understanding the different driving forces behind employees’ motivation to stay in an organization (Mansour et al., 2021). In a collectivist cultural context, normative commitment may dominate because this culture strongly emphasizes loyalty and conformity. In contrast, in cultures that emphasize personal fulfillment and self-actualization, affective commitment may be more influential (Nauman et al., 2021). These differences in the impacts of environmental control and cultural configurations suggest that organizations must develop specific commitment-building strategies according to their particular workforce compositions. If an employee has an affective commitment to her organization, she may choose to stay due to emotional factors, which further blurs the boundary between affective commitment and normative commitment (Shoaib et al., 2021). However, despite these criticisms, the three-component model remains the primary framework for studying organizational commitment and its antecedents.

### 2.2 The role of training in human resource development (HRD)

Training is a vital part of human resource development (HRD), enhancing an organization’s human capital. Employee training is crucial for HRD strategies, as it improves employee performance, satisfaction, and long-term loyalty (Al-Aali, 2021). Through training, employees gain the skills to do their jobs well, adapt to new tasks, and meet organizational goals, making this key to an organization’s success and sustainability.

Research shows that training programs impact organizational commitment directly and indirectly (Marta et al., 2021). Directly, training provides employees with skills, increases their employability and job satisfaction, and strengthens affective commitment as they sense growth in the organization (Jang et al., 2021). Organizational investment in training signals to employees that they are valued, potentially fostering normative commitment (Alolayyan et al., 2021).

Training indirectly influences commitment by enhancing job performance and promotion prospects (Ji-Young and Huang, 2021). Competent and confident employees are more loyal and satisfied. Those seeing growth opportunities are less likely to leave, strengthening continuance commitment, especially in industries with specialized skills (Bahri et al., 2021).

In addition to performance and satisfaction, training can shape organizational culture and values (Donkor et al., 2021). It communicates the organization’s mission, vision, and values, aligning individual and organizational goals. This alignment is essential for affective commitment since employees tend to identify with organizations that share their values (Al-Refaei et al., 2023). Teamwork-centered training can strengthen loyalty and normative commitment (Pathan, 2023).

However, training effectiveness in promoting commitment depends on design, implementation, and relevance. Irrelevant training has little impact, whereas training matching employees’ needs and career goals is more likely to foster loyalty (Soelton, 2023). Training timing and frequency also matter; continuous training is more effective for long-term commitment than one-time or occasional sessions (Siswadi et al., 2023).

### 2.3 Training programs and employee motivation

Employee motivation significantly impacts organizational commitment, and training programs are pivotal in inspiring employees to perform well and remain in the organization (Shahriari et al., 2023). Self-determination theory posits that individuals are motivated to engage in activities that fulfill their needs for autonomy, competence, and relatedness (Ryan and Deci, 2000). Training programs that provide employees with opportunities to acquire new skills and advance their careers can enhance their sense of competence and intrinsic motivation to work harder. Moreover, training programs focused on teamwork and collaboration can increase employees’ sense of relatedness to the organization (Silva et al., 2023).

In the context of organizational commitment, motivation can be either intrinsic or extrinsic. Intrinsic motivation drives people to engage in an activity for internal satisfaction, whereas extrinsic motivation comes from external rewards such as promotions, financial incentives, or job security (Jufrizen et al., 2023). Training programs can influence both types of motivation. For example, intrinsically motivated employees may view training as a chance to enhance their skills and achieve self-actualization, thereby increasing their affective commitment. In contrast, extrinsically motivated employees might see training programs as a means of career advancement or job security, thus strengthening their continuance commitment (Yasin et al., 2023).

Numerous studies have demonstrated the positive impact of well-designed training programs on employee commitment and motivation. Setiadi et al. (2023) reported that employees who received training reported higher levels of job satisfaction, motivation, and commitment than did those who did not. Similarly, Agustina et al. (2024) indicated that training programs not only promoted employees’ affective and normative commitment by providing opportunities for personal and professional development but also served as a powerful tool for motivating employees and fostering long-term commitment (Anand et al., 2023).

However, the relationship between training and motivation is not always straightforward. Some researchers suggest that the effectiveness of training programs in enhancing motivation and commitment depends on employees’ characteristics, such as their learning orientation and career aspirations (Purnamasari et al., 2023). For example, employees with a strong eagerness to learn and develop their skills can benefit more from training programs than those with less motivation. Additionally, the organizational environment where training programs are carried out can influence the impact of these programs on motivation and commitment (Bankins et al., 2024). Compared with others, organizations that foster a culture of learning and development are more likely to achieve success with their training programs (Hasan and Chowdhury, 2023).

## 3 Research methodology

### 3.1 Research onion

Saunders et al. (2007) “research onion” is a widely known framework of how researchers create their research methodology. The model consists of six layers, each representing a crucial aspect of the research process: research philosophy, research approach, research strategy, time horizon, data collection methods, and techniques for data analysis. This study aims to understand these layers to select the right methods. To explain the choices made throughout this chapter, the “research onion” will be used as a framework.

### 3.2 Research philosophy

Research philosophy consists of the beliefs and assumptions that inform research methods of understanding knowledge and reality. The chosen research philosophy for this study is interpretivism. The root of interpretivism is that social reality is subjective, and human interpretation and interaction are used to construct reality (Nimran et al., 2024). For this study, interpretivism is suitable on its own accord, as the goal is to understand the notions of the lived experiences and perceptions of employees as a result of training programs on organizational commitment. The major advantage of interpretivism is its provision of greater depth into the meaning and individual perspectives associated with complex social phenomena, such as organizational commitment. Interpretivism, in contrast to positivism, which aims to uncover objective truths through empirical observation, centers around the meaning that the individual associates with his experiences. This study takes an interpretive approach to capture the complicated and subjective nature of how employees perceive training programs and their effect on organizational commitment. Delving into taking the participants’ experience, a qualitative method is the right methodology to address.

### 3.3 Research approach

Second, the research approach is the second layer of the research onion. In this study, an inductive approach is adopted, one that involves developing theory from observations and patterns found within the data. Qualitative research is particularly suited to an inductive approach, as under this approach, there will be no preconceived hypotheses as to what can be expected from the research (Lestari et al., 2024). This study will then use the inductive approach to better understand how the training effect on employee programs influences organizational commitment through participants’ experiences. Rather than testing one’s *a priori* theory, data generated from this approach yield themes that can be used to theorize about the determinants of training and commitment. This technique fits into the thematic analysis, where themes are organically seen from the data. The difference between the inductive and deductive approaches is that the latter constructs hypotheses from existing theories. Given that the research is interested in exploring employee experiences rather than testing a theory, an inductive approach offers more promise.

### 3.4 Research strategy

Selecting the research strategy is the third layer of the research onion. Using a simple case study strategy, this study explored the role of training in a particular organization. The case strategy is a research strategy that investigates a contemporary phenomenon in its real-world context (Onur et al., 2024). It permits the researcher to obtain substantial, in-depth elaborations on complex matters such as employees’ perceptions of how training affects their organismic commitment. Considering the research’s aim, this case study strategy is especially appropriate, as it allows observation of how training programs are run in the organization and how they affect employees’ attitudes, behaviors, and commitment levels. The case study focuses on a specific organizational context and thoroughly explores and compares employees’ experiences with training. Another reason for adopting the case study strategy is the scope of the study. This allows multiple data sources, such as interviews, organizational documents, and observations, to paint the whole picture of the studied phenomenon. This is particularly useful for examining, in a multifaceted way, the effect of training on commitment.

The participants were selected through purposive sampling, ensuring diversity in terms of industry, years of experience, and job roles. This was done to ensure that the findings could be generalized across different organizational contexts. Additionally, participants were chosen on the basis of their participation in recent employee training programs, making the sample representative of those directly impacted by such initiatives.

### 3.5 Time horizon

The fourth layer of the research onion addresses the time horizon of the investigation. The focus of the study is the cross-sectional time horizon, effectively taking the data at only one point in time. In qualitative research, cross-sectional studies can be used when there is a focus on understanding participants’ perceptions or experiences at a point in time (Bashar et al., 2024). This applied to the current study since the goals are to record present experiences and points of view from workers in determining the influence of training programs on organizational commitment (while a longitudinal time horizon might hold valuable insights into how training programs affect commitment over time, the scope of this study is to explore immediately, through perception and experience, employees’ immediate perceptions and experiences). Time constraints favor the cross-sectional approach because answering the research questions is feasible.

### 3.6 Data collection methods

The fifth layer of the research onion relates to the methods by which the data are collected. The primary method of data collection in this study was semistructured interviews. Qualitatively, a semistructured interview grants flexibility to explore the topic while focusing on the main themes and topics. Semistructured interviews were conducted, averaging 45 min each. The interview guide consisted of open-ended questions exploring the perceived value of training programs, career development, and organizational loyalty (see Appendix I for specific questions). The interviews allowed flexibility for participants to share their detailed experiences. In-depth discussions are created, which permit the researcher to examine how training programs affect organizational commitment as the participants perceive it (Obeng et al., 2024). This interpretive philosophy and inductive approach suggest that semistructured interviews hold promise in terms of the informativeness of the data garnered and the details provided, allowing participants cadence to talk in their own words and with their voices. The interview questions will be open-ended so that people can expand on what they experienced and what they think to conjure what they believe training programs are like. Participants will be selected through a purposive sampling strategy, where people will participate in employee training programs inside the organization. Purposive sampling selects people most likely to provide relevant and insightful information (Al-Refaei et al., 2024). The data will be satiated until no new themes or insights emerge. This is because it guarantees that the data collected are rich and detailed.

## 4 Data analysis techniques

The final layer of the research onion involves data analysis techniques. For this study, thematic analysis, as described by Braun and Clarke (2006), will be employed to analyze the data collected from the interviews. Braun and Clarke (2006) proposed a comprehensive framework for thematic analysis, which has been widely used in qualitative research. Their work provides a solid foundation for our analysis in this study. Data analysis followed a thematic approach, using NVivo software to facilitate the coding process. The coding was reviewed by two independent researchers to ensure consistency. Any discrepancies were discussed and resolved through consensus, ensuring the reliability of the thematic analysis. Thematic analysis is a qualitative data analysis method that involves identifying, analyzing, and reporting patterns (themes) within the data (Yousf and Khurshid, 2024). It is a flexible approach that allows for a deep exploration of the data while remaining grounded in participants’ experiences.

The thematic analysis process will follow six key steps:

Familiarization with the data, where the researcher transcribes and reviews the interviews.

Initial coding involves identifying key data segments relevant to the research questions.

Searching for themes involves grouping similar codes into broader categories.

The themes are reviewed, and the researcher refines them to ensure that they accurately represent the data.

Defining and naming themes involve articulating each theme’s meaning.

Writing the results, where the researcher presents the themes and supporting evidence from the data.

Thematic analysis is well suited to this study because it allows for the emergence of themes directly from the data, which aligns with the inductive approach. It enables researchers to explore how employees perceive the impact of training on their organizational commitment and to identify commonalities and differences in their experiences (Braun and Clarke, 2006). However, while thematic analysis is a flexible and useful method, it also has several limitations. For instance, the interpretive nature of the method means that different researchers may identify and interpret themes differently. To address this, we followed the rigorous six-step process outlined by Braun and Clarke and engaged two independent researchers to review the coding. This collaborative approach aimed to minimize potential biases and ensure the reliability of our analysis, balancing the method’s inherent subjectivity with structured rigor.

1. Data analysis and results
2. Thematic analysis process
3. Familiarization with the data

Phase 1: Familiarizing yourself with your data. This phase involves transcribing the interview audio recordings (if necessary), which is not only a process of converting spoken words into written form but also an opportunity to start engaging with the data. After transcription, the researcher read and reread the data carefully. During this reading process, the researcher actively searches for meanings, patterns, and potential themes within the data, immersing themselves in the content to gain a deep understanding of the participants’ experiences, as suggested by Braun and Clarke (2006).

### 4.1 Generating initial codes

To help achieve this goal, the research question’s subject was initially coded to ascertain key phrases, concepts, and ideas. Different segments of the data were assigned codes (e.g., describing the experience of training programs, thoughts on organizational commitment, and perception of the link between these two factors). The common codes that frequently emerged were “skill development,” “loyalty,” “motivation,” “career growth,” “job satisfaction,” “relationship with management,” and “sense of belonging.” As Braun and Clarke (2006) suggested, when generating initial codes, researchers should code for as many potential themes/patterns as possible, considering the diverse aspects of the data. This helps ensure that no important information is overlooked and that the analysis is comprehensive.

### 4.2 Searching for themes

The initial codes were grouped into broader categories representing significant themes related to the research question. The following main themes emerged from the data:

1. Perceived value of training programs
2. Impact on skills and career growth
3. Relationship between training and job satisfaction
4. Influence of training on organizational loyalty
5. Training as a tool for employee engagement

Each of these themes was supported by several subthemes, which provided further granularity and depth to the analysis.

Braun and Clarke (2006) emphasized that during this stage, it can be helpful to use visual representations such as tables or mind maps to sort codes into potential themes. This aids in visualizing the relationships between different codes and identifying overarching themes more effectively. We followed this approach by creating thematic maps and iterative tables to refine the connections between codes and finalize the themes.

### 4.3 Review and defining themes

The identified themes were reviewed to ensure that they accurately represented the data. Each theme was checked against the coded data and the research objectives to confirm relevance. Once the themes were refined and verified, they were named and defined in preparation for the final write-up of the analysis.

### 4.4 Thematic analysis table

After a comprehensive analysis, the main themes, subthemes, and key findings emerged, as shown in Table 1. These themes and findings are based on an in-depth analysis of the interview data and reflect employees’ perceptions of training programs, the impacts of training on various aspects of employees, and the relationship between training and organizational commitment in multiple dimensions. These findings provide an important basis for drawing subsequent research conclusions.

**Table 1:** Summarizes the data analysis’s main themes, subthemes, and key findings.

Main themes	Subthemes	Findings
Perceived value of training programs	Relevance of training content	Employees emphasized that training programs must be relevant to their job roles and career goals.
	Quality of trainers	The expertise and delivery style of trainers significantly affected the perceived value of training.
	Practical application of skills	Participants valued training with a clear, practical application in their daily tasks.
Impact on skills and career growth	Personal development	Employees believed that training programs contributed to their personal growth and skill enhancement.
	Promotion opportunities	Training was seen as a pathway to promotion and career advancement.
	Knowledge sharing	Participants mentioned that training programs fostered a culture of knowledge sharing.
Relationship between training and job satisfaction	Improved job performance	Training programs enhanced employees’ job performance, leading to higher job satisfaction.
	Increased motivation	Employees felt more motivated after attending training programs, particularly when they saw tangible benefits in their work.
	Reduced job stress	Training helped employees feel more confident in their roles, reducing work-related stress.
Influence of training on organizational loyalty	Sense of loyalty	Many employees reported that training programs increased their loyalty to the organization.
	Normative commitment	Some employees felt obligated to remain with the organization after receiving training.
	Affective commitment	The training helped foster emotional attachment to the organization.
Training as a tool for employee engagement	Interaction with management	Training provided employees with opportunities to interact with management, which boosted engagement.
	Sense of belonging	Employees felt more connected to the organization after attending training programs.
	Enhanced team collaboration	Training programs promoted team-building and collaboration among employees.

### 4.5 Themes and findings

#### 4.5.1 Perceived value of training programs

The first major theme identified in the data were the perceived value of training programs. The participants frequently mentioned the importance of training content’s relevance to their specific job roles and career aspirations. For example, one participant emphasized, “If the training does not relate to what I do daily, it feels like a waste of time.” Another participant also expressed a similar view, saying, “I took a training course last month, but most of the content was too theoretical and had nothing to do with my actual work tasks. It was truly a disappointment.” These statements clearly illustrate the importance of training content, which is crucial to the perceived value of training programs, in the eyes of employees. This sentiment was echoed by several other employees, who emphasized that training must address their immediate job needs for it to be perceived as valuable. In addition to the content, the subtheme quality of trainers was very important. The trainers played an important role in the employees’ perceptions of the total quality of the training programs. Employees said that the trainers’ delivery style and level of expertise contributed significantly. “One participant said the trainer was ‘very good’ and ‘very knowledgeable,’ which made the training ‘more engaging and useful.’” The Practical Application of Skills (Skills Application) subtheme was another in which employees favored training programs that readily gave them skills that could be immediately applied in their jobs. Practical, hands-on training sessions had a more lasting impact on site than theoretical or generic sessions did.

#### 4.5.2 Skills and career growth impact

The impact on skills and career growth was dominant in all interviews. The training programs were important contributors to employees’ personal and professional development. Many employees derived a personal development subtheme, saying that they felt more confident in their abilities upon training. “I was a lot more confident after the training, and I was able to use these skills, which have helped grow me in the company,” said one employee. A second key finding was the link between Promotion Opportunities and training programs. First, people saw training as a means of advancing their careers in the organization. Someone said, “The company has done a good job offering training opportunities, which helped me advance in the ranks.” Another emerging subtheme was knowledge sharing, and in the areas of this subtheme, employees reported that training programs promoted the culture of collaboration and knowledge exchange in the organization. Sharing what they had learned with their peers was encouraged, which in turn encouraged a more open and collaborative work environment.

#### 4.5.3 Relationship between training and job satisfaction

Another important theme was the relationship between training and job satisfaction. The participants indicated that the training programs put them in a quandary between job performance and job satisfaction. One example was when a person said, “After the training, I thought I was so much better at my job, and that made me feel a lot happier at what I did.” Increased motivation was closely related to job satisfaction, one of the subthemes. When they did, employees found training programs more motivating and energizing, and these employees tended to apply what they learned to their daily tasks sooner. One said, “Training gave me a little extra motivation because I could see it making me better at what I do.” The reduced-ob stress subtheme was based on the answers of the employees, who reported feeling more confident and less stressed after training.

#### 4.5.4 Influence of training on organizational loyalty

Another prominent theme from the data relates to the influence of training on organizational loyalty. Employees also said that participating in training programs increased their loyalty to the organization. Another participant said, “Having the company do something to develop our skills makes me more loyal to them.” Normative commitment and affective commitment were also defined as subthemes. Normative commitment is shown by some employees who feel that they should remain with the organization after training. “There was not much I could do,” one employee said. “I feel like the company has put a lot into my growth, so I owe it to them to stay and give back.” However, many others exhibited affective commitment, such as those who felt emotionally connected to the organization because of the support and development opportunities they received. “I do love working here,” said a participant. The training programs have helped with the feeling of attachment.ended training programs constitute another subtheme. Training, they added, led to a stronger feeling.

#### 4.5.5 Training as a way to engage employees

Training as a tool for employee engagement was the final theme that shed light on how such training programs act as an engagement tool that helps engage employees with the organization at a deeper level. One such subtheme is interaction with management, wherein participants noted that the training programs often facilitated interaction with senior management, making them feel more valued. One interviewee mentioned that they had training sessions that also took place with upper management and that made them feel more connected to the company.

The consistent sense of belonging after people attended training in the organization. Some say that after the training, I felt a bit more like I was part of the team, which encouraged me to work longer with the company. The subtheme of Enhanced Team Collaboration was finalized. Employees report that training programs increase collaboration and teamwork among their colleagues. That training helped us work better as a team, and that is good for the department.

#### 4.5.6 Interconnections among themes

The themes identified in this study form a dynamic, interrelated framework that illustrates how employee training programs holistically shape organizational commitment. As outlined in Table 1, these themes—Perceived Value of Training Programs, Impact on Skills and Career Growth, Relationship between Training and Job Satisfaction, Influence of Training on Organizational Loyalty, and Training as a Tool for Employee Engagement—intersect to create a compounding effect on employee engagement and loyalty.

For example, the theme Perceived Value of Training Programs (with subthemes Relevance of Training Content, Quality of Trainers, and Practical Application of Skills) directly feeds into Impact on Skills and Career Growth (Personal Development, Promotion Opportunities, and Knowledge Sharing). Employees who viewed training as relevant to their roles and delivered by knowledgeable trainers (subthemes under perceived value) were more likely to recognize its contribution to their personal development and career advancement. As one participant noted, “The training I received was exactly what I needed for my current job. It gave me new skills I could apply right away, and I feel more confident in my ability to grow here. “This alignment between practical skill application (a subtheme of perceived value) and career growth (a subtheme of impact on skills) reinforces employees’ belief in organizational support for their advancement.

Similarly, the relationship between training and job satisfaction (improved job performance, increased motivation, and reduced job stress) intersects with the influence of training on organizational loyalty (sensity of loyalty, normative commitment, and affective commitment). Enhanced performance and reduced stress (subthemes of job satisfaction) fostered emotional attachment (affective commitment) and a sense of obligation (normative commitment) to stay with the organization. One participant explained, “After training, I felt less overwhelmed and more capable. It made me want to stay longer because the company cares about my growth. “Here, reduced stress (a job satisfaction subtheme) bridges skill development and loyalty, illustrating how training alleviates workplace challenges while strengthening commitment.

The theme Training as a Tool for Employee Engagement (Interaction with Management, Sense of Belonging, Enhanced Team Collaboration) amplifies the effects of other themes. For example, opportunities to interact with leadership during training (an engagement subtheme) strengthened employees’ sense of belonging, which further enhanced their perception of training value. One employee highlighted, “Training sessions with senior leaders made me feel valued. I now see my role as part of a bigger mission, which keeps me motivated. “This interaction (an engagement subtheme) not only boosted motivation (a job satisfaction subtheme) but also reinforced affective commitment (an organizational loyalty subtheme).

Collectively, the data in Table 1 demonstrate that training programs act as multifaceted interventions. By addressing practical skill gaps (via the relevance of content), fostering career aspirations (through promotion opportunities), and nurturing social dynamics (via team collaboration), organizations create a reinforcing cycle of engagement, satisfaction, and loyalty. To maximize impact, training design must integrate these dimensions—ensuring alignment between content relevance, developmental goals, and opportunities for meaningful interaction.

## 5 Discussion

This research aimed to investigate the role of employee training programs in enhancing organizational commitment through thematic analysis of qualitative data from semistructured interviews. The training program shaped employees’ perceptions, attitudes, and behaviors toward the organization and promoted organizational commitment. Perhaps the study’s most significant finding was the value of training programs as perceived by respondents. In particular, employees continued to discuss the degree to which training content was relevant to their job roles as a key factor contributing to the program’s effectiveness. This finding is consistent with prior research indicating that training will better engage employees when they feel that training is practical and applicable to the tasks they perform every day (Abu Orabi et al., 2024). Finally, the quality of trainers and the practical application of skills learned through training were the most critical factors influencing how participants assessed the overall quality of the programs. This implies that organizations must pay special attention to the relevance of training and how it is delivered to achieve maximum employee effect.

The research also explored how training improves employees’ skills and helps them grow their careers. Many participants viewed the training as the means to improve their competencies and move up in the organization. This aligns with findings demonstrating a strong relationship between employee development opportunities and career advancement (Buga, 2024). Furthermore, through this thematic analysis, training programs encouraged knowledge sharing among employees and enhanced a collaborative work environment. This finding indicates that training programs can facilitate the organization’s culture of learning and cooperation, which is critical for organizational growth and innovation. This study also revealed that training leads to a direct link with job satisfaction. The training programs were reported to improve employees’ job performance, which, in turn, increased employees’ job satisfaction and motivation. This finding is consistent with Herzberg’s two-factor theory, which suggests that personal growth and development opportunities are important motivators for employee satisfaction (Noesgaard and Jørgensen, 2024). Moreover, the training reduced job stress for employees by increasing their confidence in their roles. This shows that you need to train and develop your employees continuously to retain their productivity and satisfaction.

The continuity of training and its influence on organizational loyalty constitute another theme. The training programs were generally cited by many of the participants as raising the level of their feeling of loyalty to the organization, especially when they perceived that the organization had invested in their development. This is consistent with social exchange theory, which states that rational workers are more likely to reciprocate with higher levels of commitment if they perceive that their organization is committed to their growth (Taamneh et al., 2024). In addition, the study detected both affective and normative commitment among employees, which indicates that training programs may also create emotional and organizational obligations for the organization. Finally, training programs are discovered to be tools for drawing workers. The results revealed that training programs offered employees the chance to interact with management and form relationships with fellow employees, thus making them more part of the organization. This makes sense in the context of the employee engagement concept, as it is an emotional and psychological link to the organization beyond job satisfaction (Ahad et al., 2021). This means that in addition to improving employees’ skills, training programs also work toward improving employees’ engagement and the latter’s sense of being part (of) a team in the organizational context.

### 5.1 Future directions

Although this study offers important suggestions regarding the contribution of employee training programs to organizational commitment, future research should address several issues. Second, longitudinal studies could test how training affects organizational commitment over time. If this is done, researchers can study the longer-term effects of training programs and determine whether the initial increase in commitment persists for months or years. For example, future studies could examine the differences across all types of training programs (e.g., leadership development, technical skills, soft skills) and their degree of influence on organizational commitment. This would offer a more fine-grained understanding of which types of training are most effective at identifying employee commitment and loyalty.

Organizational culture influences the effectiveness of training programs, and it is another area for future research. We find that organizational cultural dynamics are different, and investigating how these dynamics affect employees’ perceptions of training and how these perceptions might impact employees’ levels of commitment would be interesting. Furthermore, future studies of this nature can also investigate how employees’ personality traits, career goals, or learning preferences affect their responses to trainee programs. Understanding these factors would help organizations take different training initiatives to meet the variety of work stresses of their workforce.

### 5.2 Practical and managerial implications

This study’s implications are practical and managerial for organizations desiring to increase organizational commitment through training. First, training programs must align with employees’ organizational roles and demands. Second, training content should be aligned with an individual’s aspirations. Training content that targets the employee’s immediate activity or career progression is likely to be perceived as valuable by the employee, leading to a higher level of the employee’s identification with the organization. Third, organizations should pay attention to selecting trainers and delivering training strategies and tactics appropriately. This research revealed that employees who were knowledgeable and enthusiastic trainers trained seemed to benefit more. This means that focusing on procuring and developing highly skilled trainers and including elements that make training highly practical and engaging can improve training performance.

Another significant result is that organizations must design training interventions that require knowledge sharing. One of the principles seen as a best practice by the employees of this study was the collaborative perspective of training. Organizations should foster an environment that will make employees willing to share what they have learned with the rest. This can be done in personal counseling meetings, seminars, or even career sponsorship services that enable employees to share with others. The study also underlines the need for constant training and development to retain employees’ high levels of satisfaction with their jobs and organizational commitment. Training must also be viewed as a development process, which naturally should not be confined to a single, one-time exercise. To improve employees’ commitment to the organization, it is advisable to provide training sessions for them at least once a year, which will meet the middle of the employees’ needs in the workplace. Finally, organizations should understand the duties that training possesses in regard to engaging employees and building good rapport with upper management. Executive development initiatives allow subordinates to engage with management and acquire information about an organization’s direction and plans. Such interactions should be trained since they create feelings of belonging, increasing employee commitment.

## 6 Conclusion

This chapter aims to describe and understand the impact of training program practices on organizational commitment via a qualitative research approach and the thematic analysis method for extracting themes from data. The study’s implications showed that training contributes to an employee’s organization’s value assignment and perceived organizational support for career development, job satisfaction, organizational commitment, and employee engagement. Training programs positively correlate with increasing employee skills and productivity by diminishing job pressures and increasing organizational commitment. Therefore, this research recommends well-designed training programs as key strategic interventions for enhancing organizational commitment. Any organization that provides proper training on the most relevant and often sought-after training programs will create motivated and dedicated staff, thus improving the company’s performance over the long run. Training management and the organization should ensure that training initiatives support employees’ needs and career development plans; in this way, the organization can enhance training management for organizational commitment. The work adds to the current understanding of employee training and organizational commitment and provides practical insights to managers who want to improve their human capital development models. Nevertheless, more studies are needed to shed more light on whether training has a long-term impact on commitment and how and to what extent individual characteristics affect training.

## Data Availability

The raw data supporting the conclusions of this article will be made available by the authors, without undue reservation.

## Appendix I

Semistructured interview questions

**Table:**

Number	Semistructured interview questions
1	How would you evaluate the overall effectiveness of the training programs you have participated in?
2	How relevant is the training content to your daily work? Are there any particularly useful or useless parts?
3	What specific skills have you gained from the training, and how often do you use them at work?
4	Have the training programs helped you achieve your career goals? If so, in what ways?
5	How have the training programs affected your job satisfaction? Did they increase, decrease, or have no impact?
6	What’s the impact of training on your job stress? Has it gone up, down, or stayed the same, and why?
7	Have the training programs changed your sense of identity with the organization? If so, how?
8	Do you think the training programs have promoted knowledge sharing in the organization? What are the signs?
9	What do you think of the trainers’ professional abilities and teaching methods?
10	Among different training methods, which one do you prefer and why?
11	Do you think the training programs align with the organization’s strategic goals? If not, how should they be adjusted?
12	Has your communication and relationship with superiors changed after training?
13	Did the training programs offer chances to communicate with employees from other departments? What benefits did it bring?
14	What role do you think the training programs played in spreading and inheriting the organizational culture?
15	Have the training programs influenced your loyalty to the organization? If so, how?
